# High mobility group box-1: a potential therapeutic target for allergic rhinitis

**DOI:** 10.1186/s40001-023-01412-z

**Published:** 2023-10-12

**Authors:** Shuhua Wu, Yangyang Yu, Zhong Zheng, Qi Cheng

**Affiliations:** 1 Department of Child Otorhinolaryngology, Anhui Provincial Children’s Hospital, No. 39 Wangjiang East Road, Hefei, China; 2 Department of Function Examination Center, Anhui Chest Hospital, Hefei, China

**Keywords:** Allergic rhinitis, High mobility group box-1, Mechanism, Target

## Abstract

Allergic rhinitis (AR) is a prevalent chronic inflammatory disease of the nasal mucosa primarily characterized by symptoms, such as nasal itching, sneezing, runny nose, and nasal congestion. It has a high recurrence rate and low cure rate, with a lack of effective drugs for treatment. The current approach to management focuses on symptom control. High mobility group box-1 (HMGB1) is a highly conserved non-histone protein widely present in the nucleus of eukaryotes. It is recognized as a proinflammatory agent, and recent studies have demonstrated its close association with AR. Here, we will elaborate the role and mechanism of HMGB1 in AR, so as to reveal the potential value of HMGB1 in the occurrence and development of AR, and provide a new target for clinical research on the treatment of AR.

## Introduction

AR, also known as allergic rhinitis, is a type I allergic disease characterized by repeated sneezing, runny nose, nasal congestion and itching, accompanied by red eyes and watery eyes, and other manifestations include itchy palate and cough et al. [1]. AR not only diminishes patients' work efficiency and quality of life but also leads to increased consumption of medical resources, exerting a significant social and economic burden. In addition to common nasal symptoms, patients can also suffer from asthma [2–5]. Pawankar et al. [6] noted that approximately 400 million people suffer from AR, affecting 10–30% of the global population. Likewise, research has shown that the prevalence rates range between 10% and 30% among children and adults in the United States and other developed nations [7]. AR has emerged as a growing global concern in the fields of health, medicine, and economics [8]. The treatment of AR primarily encompasses drug therapy, immunotherapy, environmental control, and health education. Drug therapy and allergen-specific immunotherapy are the most commonly employed approaches, while environmental control and health education aim to prevent patient exposure to allergens or irritants [9]. Conventional treatment drugs for AR include glucocorticoids, antihistamines and anti-leukotrienes. Allergen-specific immunotherapy, which encompasses both subcutaneous and sublingual methods, is frequently utilized. In recent years, significant progress has been made in the prevention and treatment of allergic rhinitis. The implementation of standardized treatment measures has proven effective in controlling various symptoms and significantly enhancing patients' quality of life [10–12]. However, the long-term therapeutic efficacy remains suboptimal, highlighting the lack of effective prevention and treatment measures for AR, which contributes to the ongoing challenges in its management. Consequently, the investigation of AR pathogenesis and potential therapeutic targets has garnered increasing attention from scholars.

The high mobility group proteins were initially extracted and identified in the bovine thymus in 1973. They were named based on their rapid migration in polyacrylamide gel electrophoresis. Among these proteins, HMGB1 is a highly conserved non-histone protein that is widely present in the nucleus of eukaryotes [13]. It has been established to play a crucial role in various diseases as a proinflammatory factor [14, 15], including sepsis, tumors, atherosclerosis, tissue ischemia–reperfusion injury, arthritis, asthma, chronic nephritis, and systemic lupus erythematosus [16–20]. Under specific conditions, HMGB1 can be released from the cell and interact with various cytokines and chemokines, leading to the amplification and perpetuation of the inflammatory response [17, 21, 22]. This process mediates the occurrence and progression of inflammatory diseases. As for the role of HMGB1 in tumor, HMGB1 demonstrates dual functionalities in the context of cancer progression and treatment. Specifically, HMGB1 can promote tumorigenesis. Elevated production of HMGB1, often attributed to chronic inflammatory responses, is implicated in the onset of tumorigenesis [23, 24]. Conversely, HMGB1 exhibits a protective function in inhibiting tumor progression and enhancing the efficacy of tumor chemoradiotherapy and immunotherapy. Within the nucleus, HMGB1 facilitates the regulation of telomeres and ensures genome stability. A deficiency in HMGB1 precipitates genome instability, subsequently promoting tumorigenesis [25, 26]. In recent years, an increasing number of studies have focused on the role of HMGB1 in allergic diseases, such as asthma and AR et al. [27, 28]. Among these studies, HMGB1 and AR have emerged as the most frequently studied subjects. Here, we will focus on the pathogenesis of AR to clarify the role and mechanism of HMGB1 in the occurrence and development of AR, for the purposes of identifying potential targets for the treatment of AR.

## The pathogenesis of AR

AR is a type I allergic inflammation mediated by immunoglobulin E (IgE) [29]. Several factors contribute to the pathogenesis of AR. Multiple signal transduction pathways involved in the occurrence of AR are all related to the imbalance of Th1/Th2 cytokines, or are closely related to AR-related inflammation and immune cells, or are simultaneously associated with several factors [11, 30, 31]. Here, we will specifically clarify the pathogenesis of AR from the following three aspects.

### Nasal mucosal epithelial barrier injury

The nasal mucosa is the first line of defense of the nasal cavity against the factors of airborne infection, the dysfunction of the nasal microflora has a significant impact on the occurrence and development of nasal inflammation. Impaired epithelial barrier function facilitates allergen penetration into the lower mucosa, resulting in monocyte activation and triggering a cascade of allergic reactions [32–34]. When mucosal integrity is compromised, the mucosal epithelia produces and releases injury-related molecules that chemotaxis and activate antigen-presenting cells, thereby activating and promoting innate and adaptive immunity. Histone deacetylase (HDAC) is considered a crucial factor in allergic inflammation and tight junction dysfunction. For instance, when the nasal mucosal epithelial barrier is damaged, HDAC activates mucosal repair mechanisms and triggers a protective inflammatory response [35–37]. Furthermore, local epithelial damage to the skin and mucosal barrier can result in the release of epithelial cytokines, such as IL-25 and IL-33, which can trigger allergic reactions and contribute to the development of AR [38, 39]. Therefore, safeguarding and restoring the integrity of the epithelial barrier play a crucial role in the pathogenesis of AR.

### Antigen presentation and sensitization

AR is a chronic inflammatory disease mediated by a diverse range of immune inflammatory cells. Currently, the widely recognized immune theory posits that an imbalance in the mechanism of helper T lymphocytes (Th) plays a prominent role [40–42]. Normally, the proliferation of Th1 and Th2 cells maintains a relatively balanced state. When viruses and bacteria enter the mucosa, the body initiates a Th1 reaction, prompting Th1 cells to secrete IL-2 and IFN-γ, which mediate cellular immunity against infections. Stimulation of the airway mucosa by allergens results in excessive polarization of Th2 cells, leading to the overexpression of Th2 effector factors (IL-4, IL-5, IL-13), which mediate humoral immunity. Th2 cytokines act on B cells, inducing their transformation into plasma cells that produce and secrete specific IgE, thereby further mediating the occurrence of allergic reactions [43, 44].

### The onset of symptoms and inflammation

**Fig. 1:**
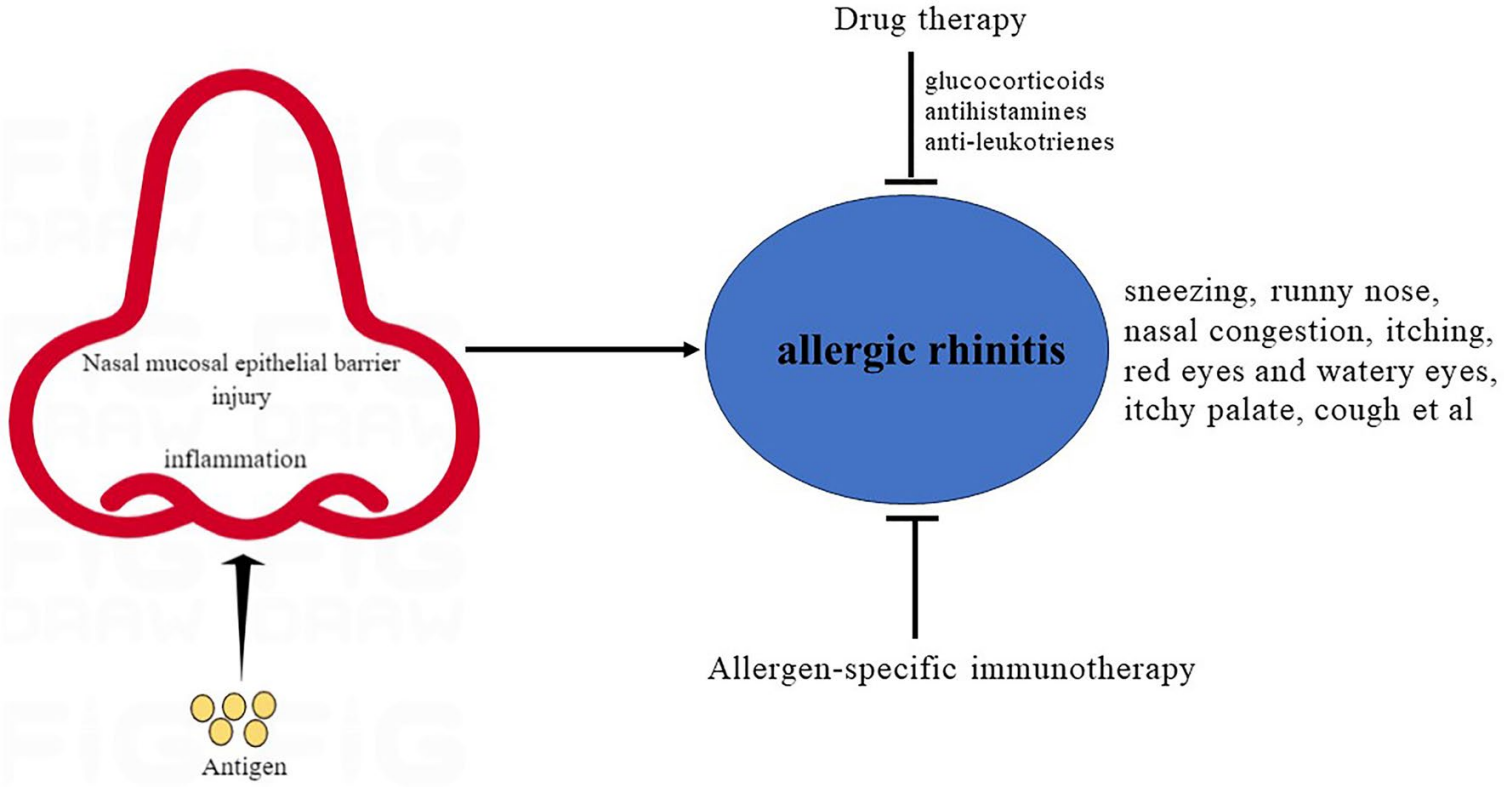
Pathogenesis, symptoms and treatment of AR. Nasal mucosal epithelial barrier injury, antigen presentation and sensitization, inflammation collectively contribute to AR, accompanied by repeated sneezing, runny nose, nasal congestion and itching, red eyes and watery eyes, itchy palate and cough et al. Drug therapy and allergen-specific immunotherapy are the most commonly employed approaches. Conventional treatment drugs for AR include glucocorticoids, antihistamines, and anti-leukotrienes

AR symptoms occur when a patient, who has been sensitized by previous exposure to an allergen, encounters the pathogenic agent again. The allergen binds to allergen-specific IgE on mast cells present in the nasal mucosa, leading to the cross-linking of IgE with FcεRI. This activation of mast cells triggers the release of both pre-stored and newly synthesized mediators, including histamine, thiopeptide leukotrienes (leukotriene C4 and leukotriene D4), prostaglandin D2, and other substances [45–47]. These active mediators bind to their respective receptors, resulting in local and systemic changes. For instance, histamine activates H1 receptors in sensory nerve endings, leading to itching, systemic reflexes, and triggering of paroxysmal sneezing. The accumulation of inflammatory mediators can result in severe allergic reaction symptoms. Furthermore, leukotrienes, vascular endothelial growth factor, prostaglandin D2, and other mediators can induce plasma exudation in blood vessels, leading to edema, blood deposition in the venous sinusoids, and increased secretion of glandular mucus. These effects can contribute to nasal congestion and the early manifestation of acute AR symptoms [6, 12, 48]. The pathogenesis, symptoms and treatment of AR were summarized, as shown in Fig. 1.

## Overview of HMGB1

High mobility group protein was first isolated from calf breast gland chromatin by Goodwin et al. Because of its solubility in 10% trichloroacetic acid, rapid migration in the polyacrylamide gel electrophoresis system, and absence of aggregation, the protein is referred to as the "high mobility group protein" or HMG protein [49]. HMGB1 belongs to the HMG subfamily. The structure of HMGB1 comprises two DNA-binding functional domains (A-box and B-box) and a C-terminal region that is negatively charged and rich in acidic amino acids [50]. The B-box region is the primary active region of HMGB1, and its release into the extracellular system promotes the release of cytokines and contributes to a pro-inflammatory response. In contrast, the A-box region lacks the pro-inflammatory properties of the B-box, but it competes with the B-box for binding sites, resulting in a dampening of the inflammatory cascade [51, 52]. HMGB1 is a crucial chromatin protein primarily located in the nucleus and widely distributed in various tissues, such as the heart, brain, liver, lymphatic system, lungs, kidneys, nasal mucosa, and others. It plays a significant role in regulating DNA stability, replication, transcription, and translation. Recent studies have shown a strong correlation between HMGB1 and various pathophysiological processes. HMGB1 primarily exerts its effects through four pathways: the receptor for advanced glycation end products (RAGE), Toll-like receptor 4 (TLR4), Toll-like receptor 2 (TLR2), and chemokine receptor 4 [53]. The pivotal role of HMGB1 in the production and regulation of inflammation has been well-established. In colorectal cancer, LPS promotes tumor formation by releasing inflammatory factors through pathways associated with HMGB1 [54]. Furthermore, researchers have discovered that HMGB1 exhibits proinflammatory effects in the treatment of infectious diseases and sepsis. They found that the prognosis of sepsis improves after administering monoclonal antibodies targeting HMGB1 without causing immunosuppression [20, 55]. HMGB1 also contributes to the regulation of inflammation in aseptic inflammation, chronic inflammation, rheumatoid arthritis, and other autoimmune diseases [20, 56]. During the inflammatory response, HMGB1 stimulates the migration of innate immune cells, promotes the innate recognition of bacterial products, activates various innate immune cells, inhibits the phagocytosis of apoptotic cells to sustain the inflammatory response, and acts as an alarm signal to further activate innate immune cells, thereby maintaining the potential for harmful inflammation [57].

## The role of HMGB1 in AR

There have been numerous studies on HMGB1 and bronchial asthma [58, 59]; however, despite the shared characteristics of AR and bronchial asthma, such as their highly reactive nature and similar pathogenesis and pathological changes, there is a dearth of research on the correlation between HMGB1 and AR. This review aims to explore the existing literature on HMGB1 and AR and elucidate the role and mechanism of HMGB1 in AR, focusing on the three aspects of AR pathogenesis mentioned earlier.

### HMGB1 leads to epithelial cell injury

Previous research has demonstrated the involvement of HMGB1 in various forms of epithelial cell injury. Evidence suggests that HMGB1 can induce acute lung inflammation, epithelial-cell barrier leakage, and even mortality in vivo [60–62]. Alveolar epithelial cells were found to exhibit HMGB1–RAGE signaling in response to LPS-induced lung injury [63]. Interestingly, Wang et al. [64] demonstrated that propofol has the potential to safeguard rats and human alveolar epithelial cells from acute lung injury induced by lipopolysaccharide by suppressing HMGB1 expression. Furthermore, HMGB1 is also implicated in renal tubular epithelial cell injury. Liu et al. discovered that downregulation of H19 suppressed HMGB1 expression, leading to the inhibition of CaOx nephrocalcinosis-induced renal tubular epithelial cell injury, NADPH oxidase, and oxidative stress both in vivo and in vitro [65]. As for the expression and function of HMGB1 in nasal epithelial cells, Chen et al. acquired epithelial cells of nasal polyps from 10 patients. They conducted an experiment where they stimulated primary cultured human nasal epithelial (HNE) cells with LPS. The results showed that LPS affects the translocation and release of HMGB1, suggesting its potential involvement in chronic rhinosinusitis with nasal polyps mediated by inflammatory factors [66]. Actually, LPS has been identified as a vital factor to induce the HMGB1 expression in nasal epithelial cells [67]. Zheng et al. conducted a study in which they obtained nasal mucosal epithelial cells from patients with nasal septal deviation and cultured them to observe the release of HMGB1 under hypoxic conditions. They employed exogenous HMGB1 to stimulate nasal mucosal epithelial cells and evaluated its impact on the permeability of fluorescein isothiocyanate-dextran 4 (FD4). In addition, they assessed the expression of ZO-1, Occcudin, Claudin-1, and E-cadherin, which are epithelial tight junction proteins. The results demonstrated a significant increase in HMGB1 release by nasal mucosal epithelial cells under hypoxic conditions. HMGB1 caused a concentration-dependent and time-dependent increase in the permeability of epithelial cells to FD4. In addition, the expression of ZO-1, Occcudin, and Claudin-1, which are epithelial tight junction proteins, was decreased, indicating compromised epithelial barrier function. This impairment increases the susceptibility of nasal mucosa to the invasion of allergens and exogenous harmful substances. These findings suggest that targeting HMGB1 may be an effective approach to intervene in nasal mucosal inflammation [68]. In another study conducted by Min et al., primary normal human nasal epithelium (NHNE) cells were cultured under hypoxic conditions. Subsequently, western blotting, immunofluorescence, and ELISA techniques were employed to evaluate the expression of HMGB1. As part of the study, the researchers also measured the level of reactive oxygen species (ROS) to investigate the translocation mechanism of HMGB1. In addition, samples of nasal mucosa and nasal lavage fluid were collected from both hypoxic and normoxic patients. The expression of HMGB1 in human nasal mucosa samples was analyzed through immunohistochemistry, while the levels of HMGB1 in lavage fluids were assessed using an ELISA assay. The findings from the study revealed that both in vitro and in vivo conditions induce the secretion of HMGB1 by nasal epithelium under hypoxic conditions. Moreover, the secretion of HMGB1 led to an upregulation of interleukin (IL)-8 production. The researchers concluded that HMGB1 secreted by nasal epithelium plays a contributory role in the inflammatory response by mediating the upregulation of IL-8 under hypoxic conditions through ROS-dependent mechanisms [69].

### HMGB1 regulates immune

HMGB1 is a protein expressed in cells and plays a vital role in regulating the immune response [16, 70], It is involved in the function of eosinophils, macrophages, and dendritic cells, among others [71, 72]. It functions as a damage-associated molecular pattern molecule, meaning it is released by cells in response to tissue damage, infection, or inflammation. Upon release, HMGB1 acts as an alarmin, alerting the immune system to danger and initiating immune responses [73]. HMGB1 can activate the innate immunity, which interacts with pattern recognition receptors (PRRs), such as Toll-like receptors (TLRs) and the RAGE, on immune cells, including macrophages, dendritic cells, and neutrophils [74]. The binding of HMGB1 to these receptors triggers intracellular signaling pathways, resulting in the activation of innate immune responses. These responses include the production of pro-inflammatory cytokines (e.g., tumor necrosis factor-alpha (TNF-α), interleukin-1 beta (IL-1β), and interleukin-6 (IL-6) and the recruitment of other immune cells to the site of inflammation [75–78]. HMGB1 also can modulate the adaptive immunity, which influences adaptive immune responses by regulating antigen presentation and T cell activation [79]. It promotes the maturation and activation of dendritic cells, which are crucial for initiating adaptive immune responses [77]. HMGB1 stimulates the expression of co-stimulatory molecules on dendritic cells, enhancing their ability to activate T cells. Furthermore, HMGB1 directly promotes the proliferation and activation of T cells, contributing to the adaptive immune response [79]. Overall, HMGB1 plays a multifaceted role in regulating immune responses. Its release and interaction with immune cells orchestrate innate and adaptive immune processes, contributing to inflammation, tissue repair, and immune defense. However, dysregulated HMGB1 signaling can also be detrimental, leading to excessive inflammation and tissue damage in various pathological conditions [80].

The imbalance of Th1/Th2 immune responses is widely recognized to play a dominant role in the pathogenesis of AR. Ma et al. [71] demonstrated that recombinant high mobility group protein (rHMGB1) exacerbates airway inflammation and mucus production in asthmatic mice. It also induces Th2 and Th17 polarization by regulating the function of dendritic cells. Conversely, antiHMGB1 was shown to impede Th2 and Th17-mediated inflammatory reactions. Another study conducted by Cavone L et al. [72] demonstrated that stimulation of the nasal mucosa by pathogens leads to the release of a significant quantity of HMGB1 into the extracellular space. Consequently, it fosters the eosinophil aggregation, thereby enhancing the Th2-mediated immune inflammatory response. Subsequently, the expression of HMGB1 is stimulated in reverse following the Th2 reaction, resulting in the production of a large number of inflammatory cytokines that further exacerbate the aggregation of eosinophils. Indeed, HMGB1 plays a crucial role in immune regulation during the pathogenesis of AR. Therefore, gaining a comprehensive understanding of the precise mechanisms underlying HMGB1 regulation and its interactions with the immune system in AR is of great interest for developing therapeutic interventions that aim to modulate immune responses.

HMGB1 is also involved in diverse inflammatory immune responses pathways. Upon exogenous invasion, immune cells actively or passively secrete HMGB1 into the extracellular space, where it binds to airway epithelial cell surface PRRs. These receptors include RAGE, certain members of the TLR family (e.g., TLR2/TLR4), and thrombospondin (TM) et al. Consequently, HMGB1 can participate in crucial inflammatory signal transduction pathways and initiate the inflammatory response [81, 82]. RAGE can be expressed in vascular smooth muscle cells, nasal mucosal epithelial cells, and other cell surfaces. It is the first receptor known to bind to HMGB1 and is considered one of the most effective receptors for HMGB1 in participating in the body's inflammatory response [83, 84]. According to Lee et al. [85], the binding of HMGB1 to the cell membrane receptor RAGE is the primary signaling pathway that triggers related inflammatory diseases. NF-κB can be translocated directly through the NF-κB pathway or indirectly through MAPK and other pathways. Moreover, this binding promotes the release of inflammatory factors, such as TNF, IL-6, and IFN-γ [16, 86], which contribute to the pathological processes of various inflammatory and immune diseases. However, the application of RAGE antibody or RAGE gene knockout methods does not fully inhibit the inflammatory response caused by HMGB1. In addition, the exact inflammatory mechanism of RAGE-mediated HMGB1 remains incompletely understood. Zhu et al. [87] observed high expression levels of HMGB1, TLR2 and TLR4 in nasal secretion samples from AR patients. However, there were no significant differences in mRNA levels of TLR3 and RAGE. They proposed that the HMGB1/TLR4 signaling pathway could serve as a potential target for AR immunotherapy. In a recent study, Yuan et al. [88] found that the HMGB1–TLR4 axis plays a crucial role in the development of AR. They also identified down-regulation of HMGB1–TLR4 as a promising therapeutic strategy to attenuate AR.

## Related inhibitors of HMGB1 in AR

Currently, there is limited availability of animal experiments and clinical trials focused on HMGB1-targeted therapy. Overall, research concerning HMGB1 in AR is nascent; however, extant studies indicate that inhibiting HMGB1 could serve as a promising and novel therapeutic approach for AR patients. HMGB1 inhibitors can be categorized into endogenous and exogenous inhibitors. Endogenous inhibitors encompass neutralizing antibodies, anticoagulants, acute phase proteins, and endogenous hormones. Exogenous inhibitors consist of herbal extracts (e.g., angelica sinensis, mung bean, and prunella sinensis) as well as herbal ingredients, such as glycyrrhizin and nicotine [89]. Ethyl pyruvate, glycyrrhizic acid, and glycyrrhetinic acid have received more attention in the context of AR studies. In the following section, we provide a brief introduction to ethyl pyruvate, glycyrrhizic acid, and glycyrrhetinic acid.

Ethyl pyruvate is a straightforward aliphatic ester derived from pyruvate and is classified as an endogenous inhibitor. Numerous studies have confirmed the efficacy of ethyl pyruvate as an HMGB1 inhibitor in various conditions, such as human malignant mesothelioma, acute kidney injury, endotoxemia, and liver injury [89–92]. Shin et al. demonstrated that ethyl pyruvate can impede the phosphorylation of HMGB1 through calcium ion chelation, preventing the nucleoplasmic translocation of HMGB1 and subsequently reducing its release [93]. Moreover, Chen et al. [94] administered ethyl pyruvate to AR mouse models, observing a substantial inhibition of Th2 cytokine expression, total IgE levels, and goblet cell proliferation in AR mice. Furthermore, ethyl pyruvate demonstrated a dose-dependent reduction in the expression and release of HMGB1.

In addition, Bhat et al. [95] discovered that ethyl pyruvate inhibits the nucleoplasmic translocation of HMGB1 induced by organic dust extract. Moreover, it decreases the expression of HMGB1 and RAGE in the cytoplasm, resulting in the alleviation of airway inflammation triggered by organic dust.

Glycyrrhizic acid and glycyrrhetinic acid are two specific compounds that are isolated from the licorice plant and belong to the group of exogenous inhibitors. Glycyrrhizic acid is utilized as an antiviral and immunomodulatory agent to prevent or treat viral infections, inflammation, and allergic reactions. It achieves this by directly binding to two BOX sites of HMGB1, which inhibits the chemotactic and mitogenic activities of HMGB1.

Glycyrrhizin, among HMGB1 inhibitors, has demonstrated the ability to reduce HMGB1 levels in the nasal fluid of patients with AR. It is effective in both adults and children and is well-tolerated compared to corticosteroids or antihistamines, without causing any adverse effects in humans [96].

Glycyrrhetinic acid exhibits anti-inflammatory and anti-allergic activities. It selectively binds to the extracellular release of HMGB1 protein and inhibits its cytokine activity by clearing the HMGB1 protein [97]. Glycyrrhetinic acid can inhibit the release of HMGB1 by up-regulating Sirt6 in cases of rhinitis [98].

## Conclusion

The identification of HMGB1 as a potential therapeutic target for AR presents exciting prospects for the future. While the field is still in its early stages, further research and development in this area hold promise for improving the management of AR and enhancing patient outcomes. Future studies should aim to deepen the understanding of the intricate mechanisms by which HMGB1 contributes to the pathogenesis of AR. This includes elucidating the specific receptors and signaling pathways involved in the HMGB1-mediated inflammatory response. By gaining a more comprehensive understanding of these mechanisms, researchers can identify new targets for intervention and develop more precise therapeutic strategies. Moreover, exploring the potential of HMGB1-targeted therapies in combination with existing treatments for AR could lead to synergistic effects and improved symptom control. Investigating the optimal timing, dosage, and administration routes for HMGB1 inhibitors in conjunction with standard anti-allergic medications may result in enhanced therapeutic efficacy. Another important aspect to consider is the development of selective and potent HMGB1 inhibitors. Currently, most HMGB1 inhibitors used in preclinical and clinical studies have shown promising results, but their specificity and potential off-target effects need to be thoroughly evaluated. Developing more specific and selective inhibitors would help minimize adverse effects and enhance the safety profile of HMGB1-targeted therapies [89]. Overall, targeting HMGB1 as a therapeutic strategy for AR is promising. Continued research, collaboration, and innovative approaches will pave the way for the development of effective and safe HMGB1-targeted therapies that can significantly improve the lives of individuals affected by AR.

## Data Availability

Not applicable.

## Abbreviations

AR: Allergic rhinitis
HMGB1: High mobility group box-1
HDAC: Histone deacetylase
Th: T lymphocytes
RAGE: The receptor for advanced glycation end products
TLR4: Toll-like receptor 4
FD4: Fluorescein isothiocyanate-dextran 4
NHNE: Normal human nasal epithelium
ROS: Reactive oxygen species
PRRs: Pattern recognition receptors

## References

[1] Jia Y, Zou J, Wang Y, Zhang X, Shi Y, Liang Y (2021). Mechanism of allergic rhinitis treated by Centipeda minima from different geographic areas. Pharm Biol.

[2] Okamoto Y, Fujieda S, Okano M, Hida H, Kakudo S, Masuyama K (2019). Efficacy of house dust mite sublingual tablet in the treatment of allergic rhinoconjunctivitis: a randomized trial in a pediatric population. Pediatr Allergy Immunol.

[3] Brozek JL, Bousquet J, Agache I, Agarwal A, Bachert C, Bosnic-Anticevich S (2017). Allergic rhinitis and its impact on asthma (ARIA) guidelines-2016 revision. J Allergy Clin Immunol.

[4] Zhang Y, Zhang L (2014). Prevalence of allergic rhinitis in china. Allergy Asthma Immunol Res.

[5] Chen Q, Shao L, Li Y, Dai M, Liu H, Xiang N (2022). Tanshinone IIA alleviates ovalbumin-induced allergic rhinitis symptoms by inhibiting Th2 cytokine production and mast cell histamine release in mice. Pharm Biol.

[6] Seidman MD, Gurgel RK, Lin SY, Schwartz SR, Baroody FM, Bonner JR (2015). Clinical practice guideline: allergic rhinitis. Otolaryngol Head Neck Surg.

[7] Schuler Iv CF, Montejo JM (2019). Allergic rhinitis in children and adolescents. Pediatr Clin North Am.

[8] Zheng Z, Yu Y (2022). A review of recent advances in exosomes and allergic rhinitis. Front Pharmacol.

[9] Eifan AO, Durham SR (2016). Pathogenesis of rhinitis. Clin Exp Allergy.

[10] Meng Y, Wang C, Zhang L (2020). Advances and novel developments in allergic rhinitis. Allergy.

[11] Bernstein DI, Schwartz G, Bernstein JA (2016). Allergic rhinitis: mechanisms and treatment. Immunol Allergy Clin North Am.

[12] Siddiqui ZA, Walker A, Pirwani MM, Tahiri M, Syed I (2022). Allergic rhinitis: diagnosis and management. Br J Hosp Med (Lond).

[13] Li Y, Xu B, Yang J, Wang L, Tan X, Hu X (2021). Liraglutide protects against lethal renal ischemia-reperfusion injury by inhibiting high-mobility group box 1 nuclear-cytoplasmic translocation and release. Pharmacol Res.

[14] Zheng H, Liang X, Han Q, Shao Z, Zhang Y, Shi L (2021). Hemin enhances the cardioprotective effects of mesenchymal stem cell-derived exosomes against infarction via amelioration of cardiomyocyte senescence. J Nanobiotechnology.

[15] Lee W, Choi HJ, Sim H, Choo S, Song GY, Bae JS (2021). Barrier protective functions of hederacolchiside-E against HMGB1-mediated septic responses. Pharmacol Res.

[16] Lotze MT, Tracey KJ (2005). High-mobility group box 1 protein (HMGB1): nuclear weapon in the immune arsenal. Nat Rev Immunol.

[17] Yang H, Wang H, Andersson U (2020). Targeting inflammation driven by HMGB1. Front Immunol.

[18] Martinotti S, Patrone M, Ranzato E (2015). Emerging roles for HMGB1 protein in immunity, inflammation, and cancer. Immunotargets Ther.

[19] Deng C, Zhao L, Yang Z, Shang JJ, Wang CY, Shen MZ (2022). Targeting HMGB1 for the treatment of sepsis and sepsis-induced organ injury. Acta Pharmacol Sin.

[20] Xue J, Suarez JS, Minaai M, Li S, Gaudino G, Pass HI (2021). HMGB1 as a therapeutic target in disease. J Cell Physiol.

[21] Bongarzone S, Savickas V, Luzi F, Gee AD (2017). Targeting the receptor for advanced glycation endproducts (rage): a medicinal chemistry perspective. J Med Chem.

[22] Andersson U, Yang H, Harris H (2018). Extracellular HMGB1 as a therapeutic target in inflammatory diseases. Expert Opin Ther Targets.

[23] Pusterla T, Nemeth J, Stein I, Wiechert L, Knigin D, Marhenke S (2013). Receptor for advanced glycation endproducts (RAGE) is a key regulator of oval cell activation and inflammation-associated liver carcinogenesis in mice. Hepatology.

[24] Yang Y, Yang L, Jiang S, Yang T, Lan J, Lei Y (2020). HMGB1 mediates lipopolysaccharide-induced inflammation via interacting with GPX4 in colon cancer cells. Cancer Cell Int.

[25] Yuan S, Liu Z, Xu Z, Liu J, Zhang J (2020). High mobility group box 1 (HMGB1): a pivotal regulator of hematopoietic malignancies. J Hematol Oncol.

[26] Mukherjee A, Vasquez KM (2020). Targeting chromosomal architectural HMGB proteins could be the next frontier in cancer therapy. Cancer Res.

[27] Li R, Wang J, Zhu F, Li R, Liu B, Xu W (2018). HMGB1 regulates T helper 2 and T helper17 cell differentiation both directly and indirectly in asthmatic mice. Mol Immunol.

[28] Ciprandi G, Colavita L, Cuppari C, Tosca MA (2023). HMGB1 modulation in children with allergic rhinitis. Minerva Pediatr (Torino).

[29] Kong Y, Hao M, Chen A, Yi T, Yang K, Li P (2022). SymMap database and TMNP algorithm reveal Huanggui Tongqiao granules for allergic rhinitis through IFN-mediated neuroimmuno-modulation. Pharmacol Res.

[30] Nur Husna SM, Tan HT, Md Shukri N, Mohd Ashari NS, Wong KK (2021). Nasal epithelial barrier integrity and tight junctions disruption in allergic rhinitis: overview and pathogenic insights. Front Immunol.

[31] Yao Y, Wang ZZ, Huang A, Liu Y, Wang N, Wang ZC (2022). T(FH) 2 cells associate with enhanced humoral immunity to SARS-CoV-2 inactivated vaccine in patients with allergic rhinitis. Clin Transl Med.

[32] Swain MS, Lebherz HG (1986). Hybridization between fructose diphosphate aldolase subunits derived from diverse biological systems: anomolous hybridization behavior of some aldolase subunit types. Arch Biochem Biophys.

[33] Karatzas K, Katsifarakis N, Riga M, Werchan B, Werchan M, Berger U (2018). New European academy of allergy and clinical immunology definition on pollen season mirrors symptom load for grass and birch pollen-induced allergic rhinitis. Allergy.

[34] Toppila-Salmi S, van Drunen CM, Fokkens WJ, Golebski K, Mattila P, Joenvaara S (2015). Molecular mechanisms of nasal epithelium in rhinitis and rhinosinusitis. Curr Allergy Asthma Rep.

[35] Islam R, Dash D, Singh R (2022). Intranasal curcumin and sodium butyrate modulates airway inflammation and fibrosis via HDAC inhibition in allergic asthma. Cytokine.

[36] Liu HL, Chen HF, Liu QD, Xu WZ, Zhang JJ, He XC (2023). HDAC Downregulation of Xiaoqinglong Decoction in the Treatment of Allergic Rhinitis. Int Arch Allergy Immunol.

[37] Zhou LB, Zheng YM, Liao WJ, Song LJ, Meng X, Gong X (2019). MUC1 deficiency promotes nasal epithelial barrier dysfunction in subjects with allergic rhinitis. J Allergy Clin Immunol.

[38] Celebi Sozener Z, Ozdel Ozturk B, Cerci P, Turk M, Gorgulu Akin B, Akdis M (2022). Epithelial barrier hypothesis: effect of the external exposome on the microbiome and epithelial barriers in allergic disease. Allergy.

[39] Celebi Sozener Z, Ozbey Yucel U, Altiner S, Ozdel Ozturk B, Cerci P, Turk M (2022). The external exposome and allergies: from the perspective of the epithelial barrier hypothesis. Front Allergy.

[40] Li P, Tsang MS, Kan LL, Hou T, Hon SS, Chan BC (2021). The immuno-modulatory activities of pentaherbs formula on ovalbumin-induced allergic rhinitis mice via the activation of Th1 and treg cells and inhibition of Th2 and Th17 cells. Molecules.

[41] Kay AB (1996). TH2-type cytokines in asthma. Ann N Y Acad Sci.

[42] Wei X, Zhang B, Liang X, Liu C, Xia T, Xie Y (2021). Higenamine alleviates allergic rhinitis by activating AKT1 and suppressing the EGFR/JAK2/c-JUN signaling. Phytomedicine.

[43] Fan Y, Nguyen TV, Piao CH, Shin HS, Song CH, Chai OH (2022). Biosci Rep.

[44] Nguyen TV, Piao CH, Fan YJ, Yu ZN, Lee SY, Song CH (2022). Artemisia gmelinii extract alleviates allergic airway inflammation via balancing th1/th2 homeostasis and inhibiting mast cell degranulation. Int J Mol Sci.

[45] Han X, Krempski JW, Nadeau K (2020). Advances and novel developments in mechanisms of allergic inflammation. Allergy.

[46] Rothenberg ME, Saito H, Peebles RS (2017). Advances in mechanisms of allergic disease in 2016. J Allergy Clin Immunol.

[47] Wheatley LM, Togias A (2015). Clinical practice. Allergic rhinitis N Engl J Med.

[48] Okubo K, Kurono Y, Ichimura K, Enomoto T, Okamoto Y, Kawauchi H (2017). Japanese guidelines for allergic rhinitis 2017. Allergol Int.

[49] Goodwin GH, Sanders C, Johns EW (1973). A new group of chromatin-associated proteins with a high content of acidic and basic amino acids. Eur J Biochem.

[50] Weir HM, Kraulis PJ, Hill CS, Raine AR, Laue ED, Thomas JO (1993). Structure of the HMG box motif in the B-domain of HMG1. EMBO J.

[51] Yang H, Ochani M, Li J, Qiang X, Tanovic M, Harris HE (2004). Reversing established sepsis with antagonists of endogenous high-mobility group box 1. Proc Natl Acad Sci U S A.

[52] Messmer D, Yang H, Telusma G, Knoll F, Li J, Messmer B (2004). High mobility group box protein 1: an endogenous signal for dendritic cell maturation and Th1 polarization. J Immunol.

[53] Thakur V, Sadanandan J, Chattopadhyay M (2020). High-mobility group box 1 protein signaling in painful diabetic neuropathy. Int J Mol Sci.

[54] Wang S, Zhang Y (2020). HMGB1 in inflammation and cancer. J Hematol Oncol.

[55] Stevens NE, Chapman MJ, Fraser CK, Kuchel TR, Hayball JD, Diener KR (2017). Therapeutic targeting of HMGB1 during experimental sepsis modulates the inflammatory cytokine profile to one associated with improved clinical outcomes. Sci Rep.

[56] Sun Y, Chen H, Dai J, Wan Z, Xiong P, Xu Y (2018). Glycyrrhizin protects mice against experimental autoimmune encephalomyelitis by inhibiting high-mobility group box 1 (HMGB1) Expression and Neuronal HMGB1 Release. Front Immunol.

[57] Wang H, Ward MF, Sama AE (2009). Novel HMGB1-inhibiting therapeutic agents for experimental sepsis. Shock.

[58] Hou C, Zhao H, Liu L, Li W, Zhou X, Lv Y (2011). High mobility group protein B1 (HMGB1) in Asthma: comparison of patients with chronic obstructive pulmonary disease and healthy controls. Mol Med.

[59] Shim EJ, Chun E, Lee HS, Bang BR, Cho SH, Min KU (2015). Eosinophils modulate CD4(+) T cell responses via high mobility group box-1 in the pathogenesis of asthma. Allergy Asthma Immunol Res.

[60] Abraham E, Arcaroli J, Carmody A, Wang H, Tracey KJ (2000). HMG-1 as a mediator of acute lung inflammation. J Immunol.

[61] Sappington PL, Yang R, Yang H, Tracey KJ, Delude RL, Fink MP (2002). HMGB1 B box increases the permeability of Caco-2 enterocytic monolayers and impairs intestinal barrier function in mice. Gastroenterology.

[62] Tang D, Shi Y, Kang R, Li T, Xiao W, Wang H (2007). Hydrogen peroxide stimulates macrophages and monocytes to actively release HMGB1. J Leukoc Biol.

[63] Zhang BF, Song W, Wang J, Wen PF, Zhang YM (2022). Anti-high-mobility group box-1 (HMGB1) mediates the apoptosis of alveolar epithelial cells (AEC) by receptor of advanced glycation end-products (RAGE)/c-Jun N-terminal kinase (JNK) pathway in the rats of crush injuries. J Orthop Surg Res.

[64] Wang X, Liu C, Wang G (2016). Propofol protects rats and human alveolar epithelial cells against lipopolysaccharide-induced acute lung injury via inhibiting HMGB1 expression. Inflammation.

[65] Liu H, Ye T, Yang X, Liu J, Jiang K, Lu H (2019). H19 promote calcium oxalate nephrocalcinosis-induced renal tubular epithelial cell injury via a ceRNA pathway. EBioMedicine.

[66] Chen D, Bellussi LM, Passali D, Chen L (2013). LPS may enhance expression and release of HMGB1 in human nasal epithelial cells in vitro. Acta Otorhinolaryngol Ital.

[67] Ciprandi G, Bellussi LM, Passali GC, Damiani V, Passali D (2020). HMGB1 in nasal inflammatory diseases: a reappraisal 30 years after its discovery. Expert Rev Clin Immunol.

[68] Zheng J, Wei X, Zhan JB, Jiang HY (2017). High mobility group box1 contributes to hypoxia-induced barrier dysfunction of nasal epithelial cells. Lin Chung Er Bi Yan Hou Tou Jing Wai Ke Za Zhi.

[69] Min HJ, Kim JH, Yoo JE, Oh JH, Kim KS, Yoon JH (2017). ROS-dependent HMGB1 secretion upregulates IL-8 in upper airway epithelial cells under hypoxic condition. Mucosal Immunol.

[70] Pisetsky DS (2012). HMGB1: a smoking gun in lupus nephritis?. Arthritis Res Ther.

[71] Ma L, Zeng J, Mo B, Wang C, Huang J, Sun Y (2015). High mobility group box 1: a novel mediator of Th2-type response-induced airway inflammation of acute allergic asthma. J Thorac Dis.

[72] Cavone L, Cuppari C, Manti S, Grasso L, Arrigo T, Calamai L (2015). Increase in the level of proinflammatory cytokine HMGB1 in nasal fluids of patients with rhinitis and its sequestration by glycyrrhizin induces eosinophil cell death. Clin Exp Otorhinolaryngol.

[73] Ulloa L, Messmer D (2006). High-mobility group box 1 (HMGB1) protein: friend and foe. Cytokine Growth Factor Rev.

[74] Li R, Zou X, Huang H, Yu Y, Zhang H, Liu P (2020). HMGB1/PI3K/Akt/mTOR signaling participates in the pathological process of acute lung injury by regulating the maturation and function of dendritic cells. Front Immunol.

[75] Branco-Madeira F, Lambrecht BN (2010). High mobility group box-1 recognition: the beginning of a RAGEless era?. EMBO Mol Med.

[76] Dyer MR, Chen Q, Haldeman S, Yazdani H, Hoffman R, Loughran P (2018). Deep vein thrombosis in mice is regulated by platelet HMGB1 through release of neutrophil-extracellular traps and DNA. Sci Rep.

[77] Ayoub M, Shinde-Jadhav S, Mansure JJ, Alvarez F, Connell T, Seuntjens J (2019). The immune mediated role of extracellular HMGB1 in a heterotopic model of bladder cancer radioresistance. Sci Rep.

[78] Li J, Kokkola R, Tabibzadeh S, Yang R, Ochani M, Qiang X (2003). Structural basis for the proinflammatory cytokine activity of high mobility group box 1. Mol Med.

[79] Khambu B, Yan S, Huda N, Yin XM (2019). Role of high-mobility group box-1 in liver pathogenesis. Int J Mol Sci.

[80] Ye Y, Zeng Z, Jin T, Zhang H, Xiong X, Gu L (2019). The role of high mobility group box 1 in ischemic stroke. Front Cell Neurosci.

[81] Takahashi H, Nishibori M (2016). Current status and future prospects in HMGB1 and receptor researches. Nihon Rinsho.

[82] Zhang Y, Karki R, Igwe OJ (2015). Toll-like receptor 4 signaling: a common pathway for interactions between prooxidants and extracellular disulfide high mobility group box 1 (HMGB1) protein-coupled activation. Biochem Pharmacol.

[83] Hori O, Brett J, Slattery T, Cao R, Zhang J, Chen JX (1995). The receptor for advanced glycation end products (RAGE) is a cellular binding site for amphoterin. Mediation of neurite outgrowth and co-expression of rage and amphoterin in the developing nervous system. J Biol Chem.

[84] Tang SC, Wang YC, Li YI, Lin HC, Manzanero S, Hsieh YH (2013). Functional role of soluble receptor for advanced glycation end products in stroke. Arterioscler Thromb Vasc Biol.

[85] Lee W, Ku S, Yoo H, Song K, Bae J (2014). Andrographolide inhibits HMGB1-induced inflammatory responses in human umbilical vein endothelial cells and in murine polymicrobial sepsis. Acta Physiol (Oxf).

[86] Park JS, Arcaroli J, Yum HK, Yang H, Wang H, Yang KY (2003). Activation of gene expression in human neutrophils by high mobility group box 1 protein. Am J Physiol Cell Physiol.

[87] Zhu X, Cong J, Yang B, Sun Y (2020). Association analysis of high-mobility group box-1 protein 1 (HMGB1)/toll-like receptor (TLR) 4 with nasal interleukins in allergic rhinitis patients. Cytokine.

[88] Yuan Y, Liu Q, Zhao J, Tang H, Sun J (2018). SIRT1 attenuates murine allergic rhinitis by downregulated HMGB 1/TLR4 pathway. Scand J Immunol.

[89] Pellegrini L, Xue J, Larson D, Pastorino S, Jube S, Forest KH (2017). HMGB1 targeting by ethyl pyruvate suppresses malignant phenotype of human mesothelioma. Oncotarget.

[90] Liu YY, Chen NH, Chang CH, Lin SW, Kao KC, Hu HC (2019). Ethyl pyruvate attenuates ventilation-induced diaphragm dysfunction through high-mobility group box-1 in a murine endotoxaemia model. J Cell Mol Med.

[91] Wagner N, Dieteren S, Franz N, Kohler K, Mors K, Nicin L (2018). Ethyl pyruvate ameliorates hepatic injury following blunt chest trauma and hemorrhagic shock by reducing local inflammation, NF-kappaB activation and HMGB1 release. PLoS ONE.

[92] Seo MS, Kim HJ, Kim H, Park SW (2019). Ethyl pyruvate directly attenuates active secretion of HMGB1 in proximal tubular cells via induction of heme oxygenase-1. J Clin Med.

[93] Shin JH, Kim ID, Kim SW, Lee HK, Jin Y, Park JH (2015). Ethyl pyruvate inhibits HMGB1 phosphorylation and release by chelating calcium. Mol Med.

[94] Chen S, Wang Y, Gong G, Chen J, Niu Y, Kong W (2015). Ethyl pyruvate attenuates murine allergic rhinitis partly by decreasing high mobility group box 1 release. Exp Biol Med (Maywood).

[95] Bhat SM, Massey N, Karriker LA, Singh B, Charavaryamath C (2019). Ethyl pyruvate reduces organic dust-induced airway inflammation by targeting HMGB1-RAGE signaling. Respir Res.

[96] Bellussi LM, Cocca S, Passali GC, Passali D (2017). HMGB1 in the pathogenesis of nasal inflammatory diseases and its inhibition as new therapeutic approach: a review from the literature. Int Arch Otorhinolaryngol.

[97] Zhang H, Yang N, Wang T, Dai B, Shang Y (2018). Vitamin D reduces inflammatory response in asthmatic mice through HMGB1/TLR4/NF-kappaB signaling pathway. Mol Med Rep.

[98] Chen D, Bellussi LM, Cocca S, Wang J, Passali GC, Hao X (2017). Glycyrrhetinic acid suppressed hmgb1 release by up-regulation of Sirt6 in nasal inflammation. J Biol Regul Homeost Agents.

